# Stress-engineered growth of homoepitaxial GaN crystals using hydride vapor phase epitaxy

**DOI:** 10.1039/c8ra06438e

**Published:** 2018-10-17

**Authors:** Moonsang Lee, Sungsoo Park

**Affiliations:** Korea Basic Science Institute 169-148, Gwahak-ro, Yuseong-gu Daejeon Korea Republic of Korea lms1015@kbsi.re.kr; Department of Science Education, Jeonju University 303 Cheonjam-ro, Wansan-gu Jeollabuk-do Republic of Korea sspark@jj.ac.kr; Analytical Lab of Advanced Ferroelectric Crystals, Jeonju University 303 Cheonjam-ro, Wansan-gu Jeollabuk-do Republic of Korea

## Abstract

We report the growth of a 3.5 mm-thick bulk GaN layer using a stress-engineered homoepitaxy method without any external processes. We employ a gradient V/III ratio during the growth, which enables a 3.5 mm-thick bulk GaN layer with a smooth surface and high crystal quality to be obtained. For a constant V/III ratio of 10, the bulk GaN layer has a flat surface; however, microcracks emerge in the GaN layer. For a constant V/III ratio of 38, the bulk GaN layer has a rough surface, without microcracks. On the other hand, by decreasing the V/III ratio from 38 to 10, the structural properties of the GaN layers are successfully controlled. The higher V/III ratio in the initial growth stage leads to a rough surface, and reduced stress and dislocation density in the bulk GaN layers, while the lower V/III ratio in the second stage of the growth provides an opposite trend, confirmed by Raman spectroscopy and X-ray measurements. We expect that this study will offer a new opportunity to achieve the growth of high-crystallinity bulk GaN without *ex situ* and complicated processes.

## Abstract 2

We report the growth of a 3.5 mm-thick bulk GaN layer using a stress-engineered homoepitaxy method without any external processes.

## Graphical abstract

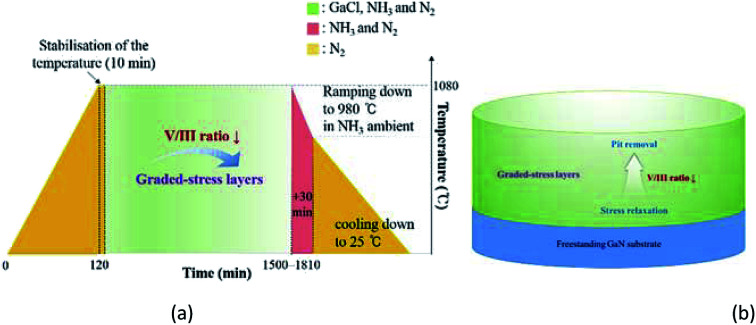

## Introduction

GaN and its compounds have been extensively studied as promising materials for opto-electrical applications such as light-emitting diodes (LEDs), laser diodes, high-power devices, and high-frequency electronics.^1–3^ Owing to the lack of native GaN substrates, conventional GaN-based devices have been hetero-epitaxially grown on foreign materials such as sapphire, GaAs, and SiC.^4–6^ However, this inevitably generates a high dislocation density (10^6^ to 10^10^ cm^−2^) owing to the difference in the lattice parameters and thermal expansion coefficient between the substrates and film, leading to lower performances of the GaN-based devices.^7^ Even if the use of a freestanding GaN substrate could overcome these challenges, the fabrication cost would be high.^8^ Low costs would be achieved by slicing bulk GaN crystals with a high crystal quality and thickness of few millimetres from freestanding GaN wafers.^9^ The growth of bulk GaN with a high crystal quality and thickness of millimetres has been investigated using various approaches, such as hydride vapour phase epitaxy (HVPE), ammonothermal, Na flux, and high-temperature high-pressure methods.^9–13^ HVPE is a practical method to grow bulk GaN crystals owing to the high growth rate and relatively good crystal quality. In the HVPE method, although bulk GaN crystals are grown on identical materials, stress can emerge in the newly grown GaN layers owing to the residual strain and lattice distortion caused by the former substrate fabrication.^14^

Surface treatment is useful for the suppression of the residual strain and lattice distortion in the growth of homoepitaxial GaN. Liu *et. al.* reported a 2 inch HVPE-grown bulk GaN wafer with a thickness of up to 3.5 mm using acid wet etching method, which reduced the strain of the newly grown homoepitaxial GaN layer.^14^ Moreover, HCl (aq), annealing in NH_3_, annealing in vapor HCl, and inductively coupled plasma (ICP) treatment are helpful to promote surface morphology for nucleation of homo-epitaxial GaN.^14–17^ These methods, however, restrict the commercial success of bulk GaN owing to the complicated growth process and the increment of fabrication cost.

In this study, we report a 3.5 mm-thick crack-free high-crystal-quality bulk GaN, obtained by controlling the V/III ratio, without any complex processes.

## Experimental

**Fig. 1:**
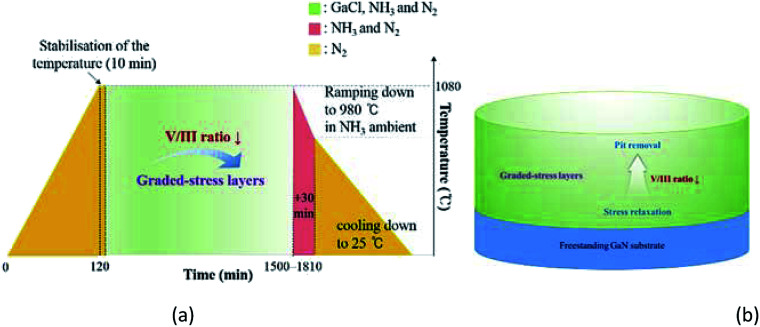
(a) Growth sequence and (b) structure of the homoepitaxial GaN layers.

The fabrication process is illustrated in Fig. 1(a). Bulk GaN was grown on a 2 inch (0001) *c*-plane freestanding GaN substrate with a thickness of 400 μm, using a vertical-type hot-wall HVPE reactor with a reactor diameter of 6 inch, under atmospheric pressure. The initial dislocation density and full width at half maximum (FWHM) of the X-ray rocking curve of the freestanding GaN substrate were approximately 2.4 × 10^6^/cm^−2^ and 123–125 arcsec, respectively. From the XRD analysis, we computed the lattice parameters of the initial freestanding GaN. The obtained values are *c* = 5.139 Å and *a* = 3.197 Å, respectively. Furthermore, E_2_ (high) peak of the material was positioned at 567.2 cm^−2^. (Not shown.) The curvature of radius was below 1 m owing to the polishing process. HCl gas was reacted with liquid Ga metal to form GaCl gas; the conversion efficiency from HCl to GaCl was ∼70%. The obtained GaCl gas was transported to the growth zone, where it reacted with NH_3_, leading to a growth of a bulk GaN layer. The flow rate of N_2_, used as the carrier gas, was 15 000 sccm. The thickness of the bulk GaN layer was 3.5 mm. In order to reduce the stress evolution and prevent a generation of pits and dislocations during the growth, a growth sequence with a gradient V/III ratio was employed. The homoepitaxial GaN obtained with a gradient V/III ratio comprises stress-relaxation, and pit-removal layers. First, a high V/III ratio is employed to reduce the stress evolution in newly grown homoepitaxial GaN layers. For this purpose, the V/III ratio was decreased from 38 to 20 at a temperature of 1080 °C. This was followed by removal of the pits generated in the previous step. For this purpose, the V/III ratio was decreased by 10. This facilitates the lateral growth of GaN, prevents the pit generation, and flattens the surface. Furthermore, the curvature radius of the bulk GaN was about 10 m. The overall structures of the homoepitaxial GaN crystals are illustrated in Fig. 1(b). In order to compare the effects of the V/III ratio, the growth conditions were varied, as shown in Table 1. The thickness of all samples was 3.5 mm. The growth rates of bulk GaN crystals with V/III ratio of 10, 38, and 38–10 were evaluated to be 99.0 μm h^−1^, 121.5 μm h^−1^, and 109.5 μm h^−1^, respectively.

**Table 1:** Growth conditions of the homoepitaxial bulk GaN layers

Condition	I	II	III
V/III ratio	10	38	38–10
Growth rate (μm h^−1^)	99.0	121.5	105.0

## Results and discussion

**Fig. 2:**
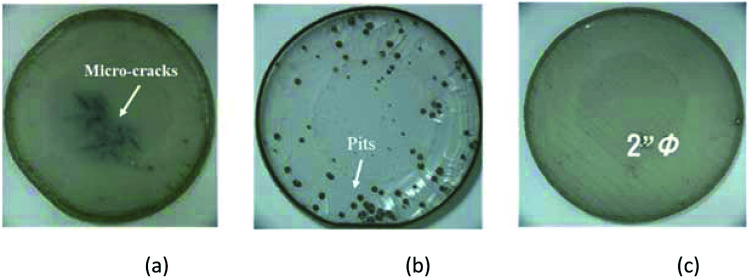
Photographs of the homoepitaxial bulk GaN samples grown using the conditions (a) I, (b) II, and (c) III.

Fig. 2 shows photographs of the 2 inch bulk GaN crystals grown using different V/III ratios. For a V/III ratio of 10 (condition I), the obtained bulk GaN had an opaque and flat surface. However, microcracks were present in the GaN layer, as shown in Fig. 2(a). These cracks were present near the surface of newly growing GaN. For a V/III ratio of 38 (condition II), the obtained bulk GaN exhibited a transparent surface and many hillocks; cracks were not observed. However, a large number of pits with large sizes were present in the GaN layer, as seen in Fig. 2(b). In order to prevent the generation of structural defects such as microcracks and pits in the bulk GaN layers, we employed a growth with a gradient V/III ratio varied from 38 to 10 (condition III). As shown in Fig. 2(c), the obtained bulk GaN with a gradient V/III ratio exhibited a smooth surface without any microcracks or large pits.

**Fig. 3:**
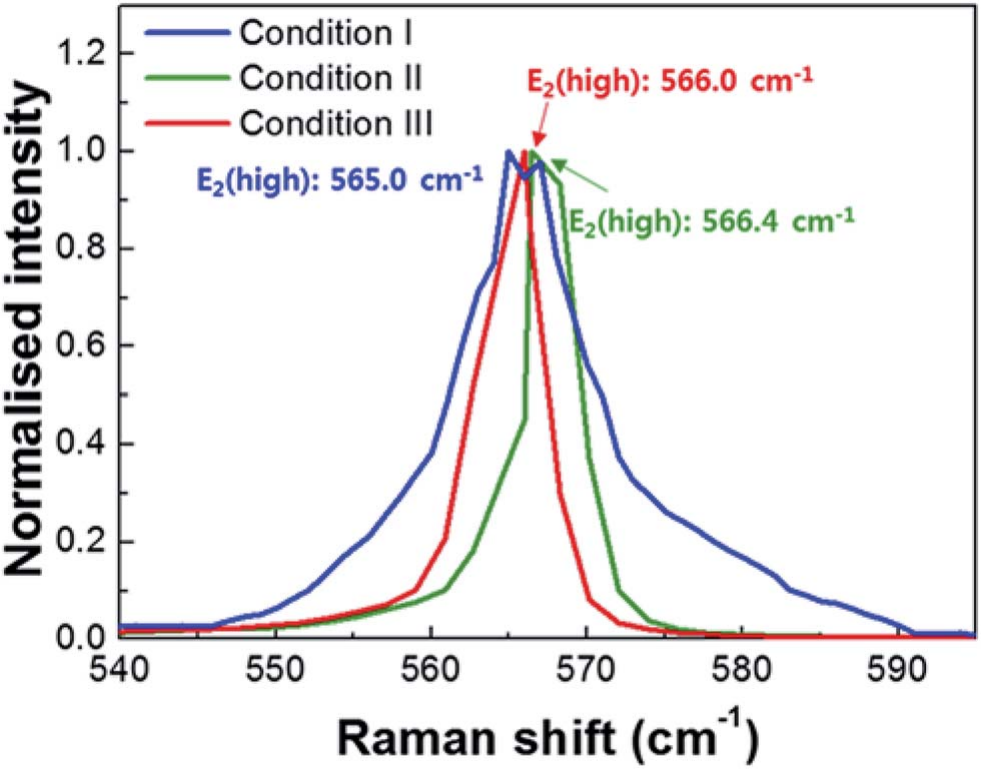
Raman spectra of the homoepitaxial GaN layers grown with various V/III ratios. The blue, green, and red curves represent the Raman spectra for the bulk GaN layers obtained using the conditions I, II, and III, respectively.

In order to understand the effects of the V/III ratio on the structural evolution of the bulk GaN crystals, we performed Raman spectroscopy measurements along the direction perpendicular to the GaN (0001) plane at room temperature, as illustrated in Fig. 3. The E_2_ (high) phonon modes of the bulk GaN crystals grown using the conditions I, II, and III can be clearly identified with the peaks at 565.0, 566.4, and 566.0 cm^−1^, respectively, indicating that the phonon frequency of the E_2_ (high) mode decreases with the decrease of the V/III ratio. It is worth noting that the peak position of the E_2_ (high) mode of a strain-free bulk GaN grown using the ammonothermal method is 568 cm^−1^.^18^ Therefore, the peak positions of the E_2_ modes are red-shifted, corresponding to residual tensile stresses of approximately ∼0.70, ∼0.37, and ∼0.47 GPa in the homoepitaxy GaN crystals, respectively. The residual stress can be calculated using the relation:^19–21^1
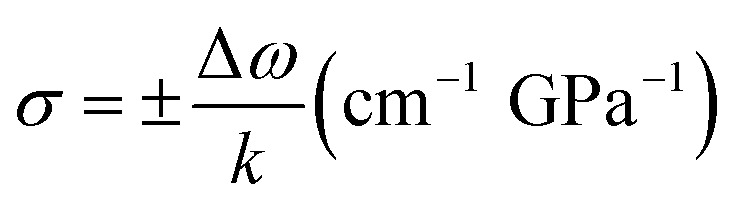
where *σ* is the biaxial stress, *k* is the linear proportionality factor, and Δ*ω* is the shift of E_2_ phonon peak.

The residual stress of the obtained bulk GaN increased with the decrease in the V/III ratio. The lower V/III ratio promoted a lateral growth, which prevented the formation of a columnar structure, thus promoting a flat surface normal to the *c*-plane. This can induce a stress accumulation in the GaN layers, which indicates that the residual stress increases with the decrease in the V/III ratio.^22^ On the other hand, a higher V/III ratio enhances the facet and the columnar growths of bulk GaN, which led to the coarse surface with many hillocks and pits. Furthermore, the facet growth leads to a reduction of the number of dislocations, owing to annihilation by bending of the dislocations towards the growth direction.^23^ These results are in an excellent agreement with the result presented in Fig. 2. We expect that an enhanced crystal quality obtained by a high V/III ratio in the initial growth stage could effectively prevent the stress accumulation in the bulk GaN layers, as the Ga-face of the substrate exhibits a high structural quality.

**Fig. 4:**
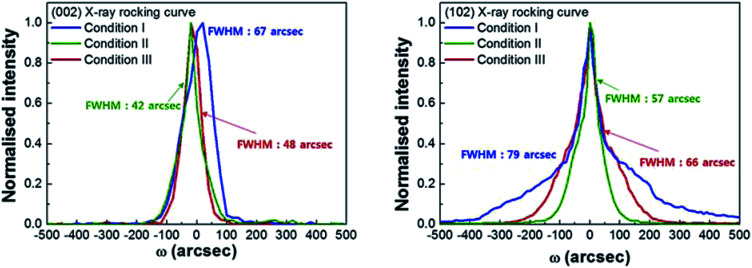
X-ray rocking curves corresponding to the (a) (002) and (b) (102) reflections of the grown bulk GaN crystals. The intensities of all curves are normalised.

In order to provide insights into the relations between the structural properties and crystal quality, we performed an X-ray diffraction analysis, as depicted in Fig. 4. The values of the FWHM of the (002) and (102) diffraction peaks in the X-ray rocking curves of the bulk GaN crystals are in the range of 42–79 arcsec, depending on the V/III ratio. The crystallinity of the homoepitaxial GaN layer improves with the increase in the V/III ratio. These results demonstrate the influence of the V/III ratio on the structural properties. A high V/III ratio leads to reduced dislocation density and stress accumulation in the GaN layers, with a rough surface. However, for a lower V/III ratio, the GaN layers exhibit high dislocation density and stress accumulation in GaN layers with a planar surface without hexagonal-shaped pits. Considering these phenomena, homoepitaxial GaN crystals with reduced stress and dislocation density, and flat surface, can be achieved using a gradient V/III ratio. The high V/III ratio in the initial stage not only prevents a stress accumulation but also it balances the crystal quality between the newly grown GaN layers and Ga-face of the substrates. Even if there are no differences in the physical properties, such as lattice constant and thermal expansion coefficient, in the homoepitaxial growth, a difference in crystal quality between the growing GaN and grown GaN layers exists, which generates a stress evolution.^24^ We expect that a high crystal quality growth in the early stage can reduce the stress generation. With the decrease in the V/III ratio during the growth, the hexagonal pits generated on the surface of the growing GaN layers cannot propagate into newly growing GaN layers, which enables to obtain a planar surface of the homoepitaxial GaN crystal. If a too low V/III ratio is employed to prevent the generation of pits in the growth stage, stress can emerge in the growing GaN layer, leading to the microcracks observed near the surface of the bulk GaN layer, as shown in Fig. 2(a). By employing appropriate V/III ratios for stress relaxation and pit generation, the generation of microcracks can be neglected. Furthermore, the lattice constants of stress-engineered bulk GaN were changed into *c* = 5.197 Å and *a* = 3.168 Å, respectively. Besides, we speculate that there are no significant change on the optical transmittance depending on the V/III ratio, considering the absence of the colour change in the bulk GaN crystals with variation of the V/III ratio.

## Conclusions

A 3.5 mm-thick homoepitaxial GaN layer was grown using a stress-engineered method without any external process. A gradient V/III ratio growth successfully offers to obtain a bulk GaN layer with a smooth surface and high crystal quality. In a constant V/III ratio of 10, the bulk GaN layer has a flat surface, accompanied by the presence of micro-cracks embedded in the bulk GaN layer. When applying a constant V/III ratio of 38 in the growth, the bulk GaN layer has a rough surface without the microcracks and the presence of pits. In a stress-engineering growth *via* a decrease in the V/III ratio from 38 to 10, the bulk GaN layers exhibits a flat surface without micro-cracks and pits. We believe that this approach will offer a simple and efficient way to achieve the growth of high-crystallinity bulk GaN without *ex situ* and complicated processes.

## Conflicts of interest

There are no conflicts to declare.

## Supplementary Material
